# Knowledge, Attitude, and Practice of Mothers towards Infant Oral Healthcare

**DOI:** 10.5005/jp-journals-10005-1553

**Published:** 2018-10-01

**Authors:** Kanika S Dhull, Brahmananda Dutta, Indira M Devraj, PV Samir

**Affiliations:** 1 Reader, Department of Pedodontics and Preventive Dentistry, Kalinga Institute of Dental Sciences, Kalinga Institute of Industrial Technology Deemed to be University, Odisha, India; 2 Professor and Head, Department of Pedodontics and Preventive Dentistry, Kalinga Institute of Dental Sciences, Kalinga Institute of Industrial Technology Deemed to be University, Odisha, India; 3 Senior Lecturer, Department of Pedodontics and Preventive Dentistry, JSS Dental College and Hospital, JSS Academy of Higher Education and Research, Mysuru, Karnataka, India; 4 Senior Lecturer, Department of Pedodontics and Preventive Dentistry, Kalinga Institute of Dental Sciences, Kalinga Institute of Industrial Technology Deemed to be University, Odisha, India

**Keywords:** Early childhood caries, Infant oral health care, Knowledge, Mothers

## Abstract

**Background:**

Among all the oral diseases, dental caries is the most common chronic disease affecting the children. Early childhood caries is one of the most severe forms of dental caries affecting the children less than 3 years. Though dental caries is preventable, not much importance has been given to the preventive aspect of dental caries. This is because of the lack of oral health education. Vertical colonization occurs from caregiver usually mother to the child. Since mother play an important role in a child’s life, their knowledge about child’s oral health will have a significant impact on the child’s oral health status. A proper knowledge for the mothers regarding infant’s oral health care will be beneficial in reducing the burden of dental caries in children. Hence a study was conducted to evaluate the mother’s knowledge, attitude and practice towards infant oral health care

**Material and methods:**

A cross-sectional study was conducted among a convenient sample of 185 first time mothers with a child aged 9 to 24 months of age visiting Department of Pediatrics and Department of Pedodontics and Preventive Dentistry. Permission to carry out the study was obtained from the Institutional Ethical Board. Data were collected using a structured questionnaire. The questionnaire used in this study was divided into two sections. The first section contained demographic details and the second section contained knowledge about primary teeth and practice of oral health care. All the participants fulfilling the inclusion criteria were enrolled. The purpose of the study and the questionnaire were explained to each participant. The questionnaire was completed and returned before leaving the clinic. The data were analyzed using Statistical Package for the Social Sciences (SPSS) version 22.

**Results:**

Total 52.5% of the mothers responded that first tooth erupt after 6 months. A total of 86.5% of the mothers were not aware of the first dental visit. 77.8% did not agree that caries causing bacteria is transferred from mother to the child; 53% mothers did not agree that night time bottle feeding causes dental caries and 78.4% disagreed that nocturnal breastfeeding can cause dental caries in children. A total of 65.4% mothers feel dental check-up is not necessary when the first tooth erupt, and 95.7% mother did not have any information on infant oral health care. 72.4% of mothers started using toothbrush and paste after all the primary teeth erupted and only 5.9% of the mother had the knowledge about proper dispensing of toothpaste for children.

**Conclusion:**

Overall knowledge and attitude of mothers towards oral health care of children is poor. Health care professionals like a gynecologist, pediatrician *Anganwadi* workers who contact first-time mothers need to be trained to disseminate appropriate infant oral health care information. The mother needs to be educated about oral health during their antenatal check up

**How to cite this article:** Dhull KS, Dutta B, Devraj IM, Samir PV. Knowledge, Attitude, and Practice of Mothers towards Infant Oral Healthcare. Int J Clin Pediatr Dent. 2018;11(5):435-439.

## INTRODUCTION

Dental caries is the most prevalent and chronic disease affecting infants and children. The carious process starts as *S.mutans,* the principal bacterial component implicated in the initiation of dental caries colonizes the oral cavity of infants/children following the eruption of primary teeth. This colonization of *S. mutans* in the infants takes place through their mothers around 2 years of age; median being 26 months, which is the window of infectivity.^1,2^ Early establishment of *S. mutans* not only affects caries prevalence but to a greater extent also the level of caries experience (deft status) in children. In a study reported by Alaluusua and Renkonen, children who harbored *S. mutans* at the age of 2 years had a significantly high Decayed missing or filled teeth surface (DMFS) score than those children who acquired this bacteria at 4 years (DMFS 10.6 *vs* 3.4).^3^

A child’s degree of colonization of cariogenic bacteria is dictated by the mother’s *S. mutans* level at the time of transmission; suggesting that mother/caregiver’s oral health status has a direct influence on child’s oral health.4,5 Moreover, since mothers play an important role in a child’s life, their knowledge about a child’s oral health will have a significant impact on the oral health status of children.

Severe early childhood caries is one of the most severe forms of dental caries affecting the children less than 3 years.^6^ National Oral Health Survey conducted throughout India has the prevalence of ECC among different states ranges from 47.5 to 78.57.^7^ Though dental caries is preventable, the burden of disease is very high. This is because of the lack of preventive oral health education. Scientific literature reveals that poor knowledge and attitude among the caregivers is the major reason for bad oral health status among the children.^8^

A proper knowledge of the mothers regarding infant’s oral health care will be beneficial in reducing the burden of dental caries in children. The American Academy of Pediatric Dentistry (AAPD) recommends the assessment of mother’s knowledge and attitude by using validated questionnaire which helps to form an effective child oral health promotion program.^9^ Hence a study was conducted to evaluate the mother’s knowledge, attitude and practice towards infant oral health care.

## MATERIAL AND METHODS

### Source of Data

This cross-sectional study was carried out in the Department of Pediatrics and Department of Pedodontics and Preventive Dentistry with an aim to evaluate the knowledge and practice of the mothers on their respective infant oral health. The research protocol of this study was approved by the Institutional Ethics committee of Kalinga Institute of Medical Sciences (KIMS), Kalinga Institute of Industrial Technology (KIIT) University, Bhubaneswar.

### Selection of Samples

- For this study, a total of 185 participants, who were first-time mothers aged 20-30 years with a child aged 9-24 months, visiting the Department of Pediatrics and Dept of Pedodontics and Preventive Dentistry were selected. Mother with the same educational level (up to 10th standard) was included in the study. This study was completed between the period of February 2017-June 2017.

### Collection of Information from the Mothers

The questionnaire was based on the literature review, pilot study, and professional experience. The questionnaire used in this study was divided into two sections. The first section contained demographic details such as the age of the mother and child; the highest educational level attained and parity. The second section contained knowledge about primary teeth, possible problems and interventions on teething, attitude towards infant oral health care and oral hygiene practices followed for their children.

All the participants fulfilling the inclusion criteria were enrolled as they presented to the clinic with their children. The purpose of the study and the questionnaire were explained to each participant and consent obtained. Each participant then completed the questionnaire (framed in local language) and returned it before leaving the clinic.

### Analysis of Data

Data were collected using a structured questionnaire. The data were analyzed using SPSS version 22. Descriptive statistics were used in reporting prevalence.

## RESULTS

Among the convenient sample of 185 mothers in the present study, 48.1% were having a male child, and 51.9% were having a female child aged 9 to 24 months. Demographic details are tabulated in Table 1. Mother’s knowledge about the eruption of first teeth, first dental visit, and early childhood caries, attitude and practice towards infant oral health care was assessed using a structured questionnaire.

All the responses are expressed as percentages. 52.5% of the mothers responded that first tooth erupts after 6 months whereas 44.3% of mothers responded as after one year (Table 2). Total 86.5% of the mothers were not aware of the first dental visit (Table 3). Total 77.8% did not agree that caries causing bacteria is transferred from mother to the child; 53% mothers did not agree that night time bottle feeding causes dental caries and 78.4% disagreed that nocturnal breastfeeding can cause dental caries in children 65.4% mothers to feel dental checkup is not necessary when the first tooth erupt and 95.7% mother did not have any information on infant oral health care (Table 4). Total of 72.4% of mothers started using a toothbrush and paste after all the primary teeth erupted and only 5.9% of the mother had the knowledge about proper dispensing of toothpaste for children (Table 5).

**Table 1:** **Table 1:** Age wise distribution of child in months [n (%)]

		*9-12 mo*		*13-24 mo*		*Total*	
Male		28(15.1)		61(33)		89 (48.1)	
Female		32 (17.4)		64 (34.5)		96 (51	
Total		60 (32.5)		125 (67.5)		185 (100)	

**Table 2:** **Table 2:** Knowledge about an eruption of the first tooth

		*Frequency (n)*		*Percentage (%)*	
After 6 months		97		52.5	
After 1 year		82		44.3	
Don’t know		6		3.2	
		185		100.0	

**Table 3:** **Table 3:** Knowledge about first dental visit

		*Frequency (n)*		*Percentage (%)*	
When 1st tooth erupts		17		9.2	
When all primary teeth erupts		8		4.3	
Don’t know		160		86.5	
		185		100.0	

**Table 4:** **Table 4:** Percentage distribution of responses regarding attitude towards infant oral health [n (%)]

		*Yes*		*No*		*Don’t know*	
Caries causing bacteria can be transmitted from mother to child		9 (4.9)		144 (77.8)		32 (17.3)	
Night time bottle feeding can cause dental caries		54 (29.2)		98 (53.0)		33 (17.8)	
Frequent and prolonged nocturnal breast feeding can also cause dental caries		9 (4.9)		145 (78.4)		31 (16.7)	
Do you have prior information about infant oral health care		8 (4.3)		177 (95.7)		–	

**Table 5:** **Table 5:** percentage distribution of responses regarding practice towards infant oral health [n (%)]

				*When 1st tooth erupts*		*After all the primary teeth erupts*		*Don’t know*	
When to start using toothbrush?				7(3.8)		134 (72.4)		44 (23.8 )	
When to start using toothpaste?				13 (7.2 )		132 (71.4)		40 (21.4)	
		Pea size		Half the length of the brush		Full length of the brush		Don’t know	
How much toothpaste should be used for children?		11(5.9 )		109 (58.9 )		5 (2.8 )		60 (32.4 )	

## DISCUSSION

Pediatric triad is a three-way interaction among the child, parents and pediatric dentists. Knowledge and attitude of parents especially the mothers towards maintaining the optimum oral health of their children during infancy plays a crucial role or future oral health status and of course for overall health and well-being.^10^

The oral health habits such as oral hygiene maintenance and a proper diet have to be established right from infancy and maintained throughout early childhood. Since mothers play as a role model for their children, assessment of their knowledge and practice is essential, as it will enable the pediatric dentists to implement an appropriate programme to improve the oral health of the child.

In our study, 44.3% mothers informed that tooth erupts after 1year and 52.5% informed that it erupts after 6 months. This suggests a variation in the eruption timing of primary teeth and the need for population-specific studies on the eruption of primary teeth.

Early dental visit results in more cost-effective treatment and a positive impact on dentists.^11^ The AAPD recommends that the child’s dentition should be seen within 6 months of 1st tooth eruption and not later than 12 months of age.^12^ This helps for early intervention and education of parents on oral hygiene, early childhood caries, and prevention of early childhood caries.^13^

Surprisingly 86% of mothers in the present study did not have the knowledge on the first dental visit for their children. This finding could be corroborated to the low education level of the mothers.

Transmission of mutans streptococcus from mother to the child is well documented^14^ and the risk of the infant developing caries increases as the levels of salivary mutans streptococcus increases in mother.^15^ Apart from salivary mutans streptococcus, mothers oral hygiene, the frequency of snaking and socioeconomic status also have an impact of colonization of caries-causing bacteria in children.^16^ Most of the mothers (77.8%) disagreed that caries promoting bacteria could be transferred from them to their respective children. This finding was in accordance with other studies where 27.2 to 41% of mothers disagreed to the fact that the mother is the primary source of transmission of caries-causing bacteria.^17^

Only 29.2% of the study population responded positively that night time bottle feeding can cause dental caries. Previous studies have reported 48% of mothers agreed on night time feeding as a cause for tooth decay^18^ Knowledge regarding nocturnal breastfeeding causing dental caries in children was significantly low (4.9%) amongst mothers in our study. However higher percentages were observed in other studies.^17,19^ Educational level might be the reason for this variation.

**Table 6:** **Table 6:** Distribution of medical and dental problems suffered by child during teething [n (%)]

*Medical problems*		*[n (%)]*		*Dental problems*		*[n (%)]*	
Fever		63 (34.1)		Gum biting		115 (62.4)	
Diarrhea		121(65.4)		Increased saliva		69 (37.6)	
Cough		11 (5.9)		Finger sucking		126 (68.3 )	
Vomiting		13 (7.)3		Gum reddening		22 (11.7)	
Rashes		2 (1)					
Irritability		34 (18.5)					
Pain		10 (5.4 )					
Disturbed sleep		13 (7.3)					

Regarding the practice oral hygiene practice for infants, 72.4% of mothers agreed that they started brushing only after all the teeth erupted. A similar finding was reported by Shivaprakash et al.^20^ ( 70%); Nagarajappa et al.^17^ (58%) and Suresh et al.^21^ (56%). In the present study, teething was misattributed to fever (34.1%), diarrhea (35.4%) vomiting (7.3%) and cough (5.9%). In accordance with most of the other studies, the present study also showed the desire to gum biting (62.4%) increased salivation (37.6%), finger sucking (68.3%) attributed to teething (Table 6)

In previous studies, Wake et al.^22^ and Owasis et al.^23^ attributed 85% while Nagarajappa et al. attributed 70% infants with fever to teething. Mothers approaching doctors for the teething problems was 76% while 15% of mother opted for home remedies and 9% for both (Table 7).

**Table 7:** **Table 7:** Treatment adopted for teething [n (%)]

Doctor’s advice		140 (76)	
Home remedy		28 (15)	
Both		17 (9)	

Oral health education and evaluation inculcated in childhood lays a foundation for lifetime free disease. This can be achieved through parents especially mothers. They play as a role model for their children hence they need to be encouraged to infuse oral hygiene habits right from infancy.

Since the study was conducted in one localized hospital and the educational level of mothers was low these results cannot be extrapolated to the general population. Hence similar studies on larger and diverse population would be more useful in implementing nationwide efficient and effective oral health programmes for infants.

## CONCLUSION

Overall knowledge and attitude of mothers towards oral health care of children is poor. Health care professionals like a gynecologist, pediatrician and *anganwadi* workers who contact first-time mothers need to be trained to disseminate appropriate infant oral health care information. Mother needs to be educated about oral health during their antenatal check-up. During their visit to the Pediatrician Oral Health Care information need to be reinforced.
